# Preimplantation Genetic Testing: A Perceptual Study From the Eastern Province, Saudi Arabia

**DOI:** 10.7759/cureus.20421

**Published:** 2021-12-14

**Authors:** Fehmida Tehsin, Fatimah H Almutawah, Hawra K Almutawah, Maryam E Alwabari, Zahra M AlSultan, Hassan S Buawadh

**Affiliations:** 1 Obstetrics and Gynecology, King Faisal University, Al Ahsa, SAU; 2 Medicine, King Faisal University, Al Ahsa, SAU; 3 Internal Medicine, King Faisal University, Al Ahsa, SAU

**Keywords:** genetic disease, g6pd, scd, sickle cell disease, pgd, kingdom of saudi arabia, eastern province, perception, pgt, preimplantation genetic testing

## Abstract

Background: Chromosomal abnormalities affect many children which lead to high rates of morbidity and mortality among them. So, preimplantation genetic testing (PGT) is an evolving technology used to detect a specific genetic disorder in embryos of a couple known to be carriers or affected by a specific mutation. Similarly, it could be used in advanced maternal age which is a high risk of chromosomal abnormalities. Although PGT is a solution for many inherited chromosomal disorders, many ethical dilemmas surround its application. Thus, the aim of this study is to evaluate the community awareness and acceptance of PGT which will eventually lead to a healthier society through disease-free babies in Eastern Province, Kingdom of Saudi Arabia (KSA).

Methodology: A qualitative cross-sectional questionnaire-based study was conducted within the population of the Eastern Province of Saudi Arabia. The questionnaire was designed in Arabic and distributed electronically through social media platforms.

Results: The study included 837 participants, whose ages ranged from 18 to 65 years with the mean age 33.5 ± 11.9 years. Good awareness and acceptance were detected among 53.7% of the old aged group (50 years or more) compared to 39.5% of the young age group. Also, 44.9% of female participants had good awareness in comparison to 34.2% of males (p=.033). Participants with a higher number of children had significantly higher awareness and acceptance of PGT. Also, 44.3% of participants who knew someone in need of assisted reproductive technology, had good awareness and acceptance levels compared to 36.9% of those who did not (p=.033).

Conclusion: The perception of Eastern Province’s Saudi citizens toward PGT is found to be low. Increasing their perception toward such technology is needed as it is known that many chromosomal abnormalities are prevalent among this population, particularly sickle cell disease. Achieving this goal will eventually lead to decrease the burden of prevalent inherited diseases. Since Saudis' opinions are almost influenced by cultural and religious points of view, care should be given to these aspects.

## Introduction

Out of 150 live births, one newborn is affected with chromosomal abnormalities and approximately 5%-7% of children die due to chromosomal defects [1]. Preimplantation genetic testing (PGT), which includes preimplantation genetic diagnosis (PGD) and preimplantation genetic screening (PGS), can precisely detect chromosomal abnormalities [2]. PGT is performed in process of in-vitro fertilization (IVF), which includes: controlled stimulation of the ovaries, oocyte retrieval, a gathering of sperms, IVF, or intracytoplasmic sperm injection, embryo biopsy, DNA testing, and finally transferring the healthy tested embryo into the uterus [3]. PGT is a technology used to detect a specific genetic disorder in embryos of a couple known to be carriers or affected by a specific mutation. PGT is a screening method for married couples who had previously affected child personally or in the family, experienced recurrent miscarriages, and want to attempt pregnancy at advanced female age [4]. The aim of PGT is to prepare the couples through proper counseling to make an informed decision about pregnancy continuation or termination based on reliable information. PGT is the only way to allow this decision to be taken before the implantation [5]. The main indications to perform PGT are advanced maternal age, recurrent miscarriages, cytogenetic and molecular diseases such as sickle cell anemia [6]. On the other hand, there are many ethical dilemmas regarding PGT such as termination of the affected fetus with late-onset disease, the cost-effectiveness of PGT in addition to the already high cost of IVF technique, ethical perspective in using PGT for non-medical indication, e.g., sex selection, religious point of view regarding termination of a viable pregnancy, and cultural tradition and beliefs [7,8].

Many studies have evaluated patients as well as high-risk groups about their acceptance of this new field of medicine in Western populations, but few studies are found conducted among Saudi society. Alsulaiman et al. in 2010 investigated the parents’ attitudes toward PGD attending King Faisal Specialist Hospital and Research Center (KFSH&RC). A total of 184 participants were divided into four groups: two groups had children affected by either a hemoglobin disorder or non-syndromic deafness while the other two groups had experience with either PGD or IVF for infertility. While parents of the first two groups with the risk for genetic conditions held positive attitudes to guarantee a healthy child, the PGD group expressed their ultimate concerns about technical limitations, and the IVF infertility group was concerned about others’ negative thoughts of IVF in society [9].

Another study reported the possible acceptance of PGD by Saudi couples following the birth of a genetically affected child with either cystic fibrosis, thalassemia, hemophilia, chromosomal translocation, sickle cell anemia, or Sakati-Nyhan syndrome. Out of 30 couples who had never heard about PGD, 11 of them (37.7%) would accept the technology, 13 (43.3%) would not accept it, and two (6.7%) were not sure about it. Given the variability of participants’ acceptance of PGD, only eight of them held favorable attitudes toward using the technology [10]. There are many studies aiming to evaluate patients as well as high-risk groups about their acceptance of this new field of medicine. However, evaluating the community understanding and acceptance of PGT is still under investigation. Such an issue is important in transferring medicine from labs into clinical practice. Therefore, our current study is aiming to evaluate community understanding and acceptance of PGT which helps to have a healthier society through disease-free babies in Eastern Province, Kingdom of Saudi Arabia (KSA).

## Materials and methods

An online questionnaire-based cross-sectional study was conducted within the Eastern Province of KSA. The required sample size was calculated to be a minimum of 776 participants to estimate an average good public perception of using PGT with 5% precision at a 95% confidence level. The final sample size of participants is 837. We employed a convenience non-probability sampling technique as a sampling method.

We use a validated questionnaire from a published study conducted by Winkelman et al. [8]. All Saudi male and female citizens living in the Eastern Province aged 18-year-old or above were included in the study. We exclude non-Saudi participants, ex-pats, and those aged under 18 years. Initially, a questionnaire was constructed in English by Winkelman et al. [8] then designed into an Arabic questionnaire and reviewed thoroughly by experts. After that, it was used and distributed electronically to the targeted population. The questionnaire contained two sections: The first one dealt with sociodemographic information, the presence of congenital/chromosomal anomalies in family or relatives, and awareness of IVF. The second section contained questions to assess the awareness and acceptance of PGT. Objectives of the study and process of PGT were explained before the start of the questionnaire and participants were informed that answering the questionnaire will be considered their consent to enroll in the study.

The study has been approved by the local Intuitional Research Board (IRB) of College of Medicine, King Faisal University, Al-Ahsa, Saudi Arabia (approval number 2020-10-65).

Data analysis

We extracted the data and then it was coded and entered into statistical software IBM SPSS version 22 (SPSS, Inc. Chicago, IL). We conducted all statistical analysis using two-tailed tests. P-value less than .05 was considered to be statistically significant. For awareness and acceptance items, each correct answer or agreement was given a one-point score and the total sum of the discrete scores of the different items was calculated. A patient with a score less than 60% of the total score (4 points) was considered to have poor awareness/acceptance while good awareness/acceptance was considered with a score of 60% (5 points or more) of the maximum or more. Descriptive analysis based on frequency and percent distribution was done for all variables including demographic data, family history genetic diseases, and awareness with acceptance items with causes of accepting or refusing PGD. Cross tabulation was used to assess the distribution of awareness/acceptance level for PGD according to the participants’ personal data. Relations were tested using the Pearson chi-square test.

## Results

The study included 837 participants, whose ages ranged from 18 to 65 years with the mean age of 33.5 ± 11.9 years. There were 559 (66.8%) female participants and 574 (68.6%) were university graduates. Four hundred and seventy-two participants (56.4%) were married. A monthly income of less than 5,000 SR was reported by 514 (61.4%) respondents. More than one-third of study respondents belonged to the healthcare system. Five hundred and eighty (69.3%) participants knew someone with a genetic disease or developmental disorder. Sickle cell disease was the most commonly reported (64.5%) genetic disease followed by G6PD (51.1%), Down syndrome (29.3%), and some other sporadic disorders (Table 1).

**Table 1:** Personal characteristics of study participants

Personal characteristics	No	%
Age in years		
18-20	114	13.6%
21-30	422	50.4%
31-40	144	17.2%
41-50	116	13.9%
> 50	41	4.9%
Gender		
Male	278	33.2%
Female	559	66.8%
Educational level		
Below secondary	29	3.5%
Secondary	234	28.0%
University/above	574	68.6%
Marital status		
Single	345	41.2%
Married	472	56.4%
Divorced/widow	20	2.4%
No. of children		
None	98	19.9%
1-2	164	33.3%
3-4	140	28.5%
5+	90	18.3%
Monthly income		
< 5,000 SR	514	61.4%
5,000-10,000 SR	144	17.2%
10,000-15,000 SR	91	10.9%
15,000-20,000 SR	48	5.7%
> 20,000 SR	40	4.8%
Your field of study or work is healthcare		
Yes	318	38.0%
No	519	62.0%
Know anyone with a genetic disease or developmental disorder		
Yes	580	69.3%
No	257	30.7%
If yes, what is that disease		
Sickle cell disease	394	64.5%
G6PDD	312	51.1%
Down syndrome	179	29.3%
Delayed growth	6	1.0%
Blindness	9	1.5%
Autism	14	2.3%
Others	42	6.9%

**Figure 1:**
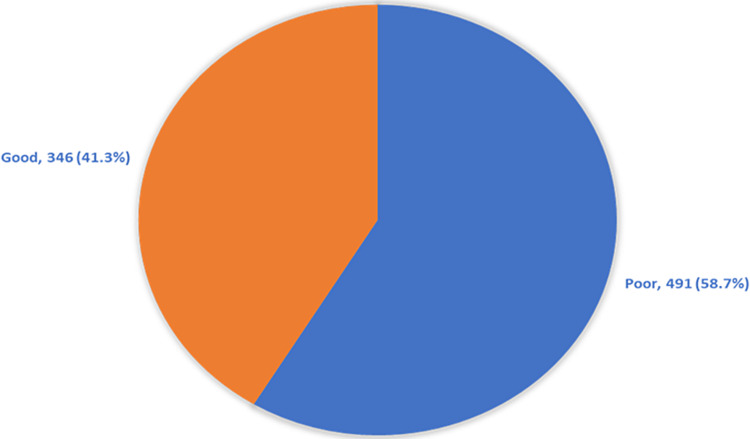
Overall participants awareness and acceptance regarding of the conduct the preimplantation genetic diagnosis

Table 2 shows participants’ awareness and perception regarding PGT. About 369 (44.1%) participants have heard about PGT. Overall participants' awareness and acceptance regarding the PGT was good among 346 (41.3%) (Figure 1).

**Table 2:** Participants awareness and perception regarding preimplantation diagnosis

PFD awareness and perception		No	%
Heard about preimplantation genetic test	Yes	369	44.1%
	No	468	55.9%
Physicians should be able to perform a genetic diagnosis prior to implantation of the embryo to detect fatal diseases in the first few years of life.	Disagree	27	3.2%
	Agree	721	86.1%
	Neutral	89	10.6%
Doctors must be able to perform a genetic diagnosis prior to implantation of the embryo to detect diseases that cause impairment of life such as mental retardation or deafness.	Disagree	29	3.5%
	Agree	730	87.2%
	Neutral	78	9.3%
Doctors must be able to perform a genetic diagnosis prior to implantation of the foetus to detect diseases that may not occur until later in life, such as diseases that put an individual at risk of developing cancer during the post-puberty period.	Disagree	102	12.2%
	Agree	561	67.0%
	Neutral	174	20.8%
Causes of accepting previous options	PGD improves the chances that a couple will have a healthy child.	618	81.7%
	PGD will lower healthcare requirement and costs	417	55.2%
	PGD can eliminate certain genetic diseases forever and may result in a better society.	568	75.1%
	It is acceptable according to the religious point of view	212	28.0%
	Others	13	1.7%
Causes of not accepting previous options	Pre-implantation genetic diagnosis reinforces discrimination against persons suffering from certain diseases.	78	33.8%
	The use of a genetic diagnosis prior to implantation of the embryo may have unfavourable consequences	71	30.7%
	It is not acceptable according to the religious point of view	123	53.2%
	It leads to unnecessary damage of embryos after detection of a genetic disease or developmental disorder	82	35.5%
	Medical errors and the possibility of any problem in replacing one sample with another	5	2.2%
	Spouses can reveal hereditary symptoms that can be passed on before marriage.	6	2.6%

Table 3 illustrates the relationship between the participants' awareness and acceptance of the PGT application and their personal data. Good awareness and acceptance were detected among 53.7% of the old-aged group (50 years or more) compared to 39.5% of the young age group with recorded statistical significance (P=.001). Also, 44.9% of female participants had good awareness in comparison to 34.2% of males (P=.033). Sixty percent of divorced/widowed participants had good awareness and acceptance for PGT compared to 46.6% of the married group and 33% of single respondents (P=.001). Participants with a higher number of children had significantly higher awareness and acceptance of PGT. Also, 44.3% of participants who know someone in need of assisted reproductive technology had good awareness and acceptance levels compared to 36.9% of those who did not (P=.033).

**Table 3:** Distribution of participants’ awareness and acceptance regarding the conduct of the preimplantation genetic diagnosis by their personal data

Personal data	Overall awareness and perception				P-value
	Poor		Good		
	No	%	No	%	
Age in years					.001*
18-20	69	60.5%	45	39.5%	
21-30	284	67.3%	138	32.7%	
31-40	69	47.9%	75	52.1%	
41-50	50	43.1%	66	56.9%	
> 50	19	46.3%	22	53.7%	
Gender					.003*
Male	183	65.8%	95	34.2%	
Female	308	55.1%	251	44.9%	
Educational level					.284
Below secondary	13	44.8%	16	55.2%	
Secondary	136	58.1%	98	41.9%	
University/above	342	59.6%	232	40.4%	
Marital status					.001*
Single	231	67.0%	114	33.0%	
Married	252	53.4%	220	46.6%	
Divorced/widow	8	40.0%	12	60.0%	
No. of children					.005*
None	57	58.2%	41	41.8%	
1-2	100	61.0%	64	39.0%	
3-4	67	47.9%	73	52.1%	
5+	36	40.0%	54	60.0%	
Monthly income					.065
< 5,000 SR	322	62.6%	192	37.4%	
5,000-10,000 SR	75	52.1%	69	47.9%	
10,000-15,000 SR	47	51.6%	44	48.4%	
15,000-20,000 SR	25	52.1%	23	47.9%	
> 20,000 SR	22	55.0%	18	45.0%	
Know anyone with a genetic disease or developmental disorder					.381
Yes	346	59.7%	234	40.3%	
No	145	56.4%	112	43.6%	
You or know someone need birth by IGSI					.033*
Yes	282	55.7%	224	44.3%	
No	209	63.1%	122	36.9%	

## Discussion

Recently, PGT is increasingly used in practice in order to have healthier babies. However, the current study indicates that it is only known to 44.1% of the respondents. Even the participants who knew someone with a genetic disease or developmental disorder (e.g., SCD, G6PD, Autism, Down syndrome, blindness) have poor awareness and perception. Despite this lack of knowledge, the attitude toward PGT was found to be good because the majority of the respondents tend to agree with PGT for medical purposes. Our study objectives and findings are similar to a study conducted in the US to evaluate the public perspective regarding PGT [8]. In that study less than one-third of their respondents were aware of the pregenetic diagnosis, the majority opted to test for fetal early and late congenital disorders. A minority of their study participants were in favor of using pre-genetic testing for non-medical reasons: personality traits, physical characteristics, and sex selection, out of which male respondents were found more keen for this use. The majority of their study participants (66.2%) believed in the main reason to support the use of PGD where couples will be able to make their own decisions regarding their babies which is similar to our participants supporting reason for the pregenetic diagnosis/testing use. Moreover, the use of these personal preferences will increase the discrimination against special groups in society and interference with nature’s law.

Another study conducted in Washington, to assess the awareness and acceptance of PGT among parents of sickle cell disease children, also revealed similar but lower awareness findings than ours, where only 24% of parents were aware of genetic testing, the vast majority of them agreed that learning about this technology is important, and will consider using PGT in the future when they would be wishing to have more children [11].

In China, a study assessed the acceptability of PGT among patients with autosomal dominant polycystic kidney disease. In that study, 60.4% of the respondents were aware that PGT technology can diagnose and modify the outcome of disease inheritance, and 79.6% of patients will choose PGT if they would plan for children in the future [12]. Recently a study has been conducted to know the awareness about genetic testing on 333 participants at King Saud University, Riyadh, Saudi Arabia, has also found a majority of their respondents (85.6%) agreed with having premarital genetic testing like our study in which the majority has displayed a positive attitude towards PGT. They had other specific genetic-based questions/scenarios for responses that are not relevant to the current study [13].

A cross-sectional study performed in Jordan on 1,111 reproductive-age women, found that 74.1% of respondents were aware of the ability of PGT to screen for genetic diseases which is far higher than the current study. The majority of their participants supported the use of PGT as a standard procedure in the national health care service especially when they know that PGT can screen and diagnose genetic abnormalities at the same time while our study did not include this aspect. [14]. Another Jordanian study that involved 463 university students demonstrated that 77% were familiar with genetic testing. The majority of the students believe that genetic testing is a useful tool to diagnose genetic diseases and, therefore, helps to prevent their occurrence. In this study, a high level of awareness was due to students’ good knowledge and exposure to medical information at different educational platforms [15].

Similarly, a recent Malaysian study conducted at Kalang Valley for the knowledge, awareness, and perception about PGT, retrieved satisfactory knowledge and affirmative perception towards genetic testing which had also shown significant association with age, ethnicity, education, study area, and with those who have heard about testing. In the current study, we found good awareness and acceptability to PGT statistically associated with old age, female participants, divorced/widow respondents, higher parity, and those who had known someone going for IVF [16].

Most of the studies have revealed remarkable awareness and acceptance of PGT. Advancement in internet technology, social media, awareness campaigns, and distance learning practices have disseminated a vast amount of pre-implantation genetic diagnosis or testing information and insight to many people all over the world. Parents with affected children, advanced aged infertility clients, and those with a family history of genetic disorders are made very well informed regarding utilizing PGT to skip disorder in their offspring by adopting preimplantation diagnosis or testing.

Most people are accepting the use of PGD because it avoids offspring suffering. This is in line with the findings of this current study. The other reason to support the use of PGD in this current study was concern about the health costs of affected children. The same concern was found by another study in which people with different hereditary cancer syndromes agreed with the use of PGD due to their beliefs that PGD will lower the overall family health costs [17]. This study indicates that the main reason behind refusing the use of PGD is religious believes which is the unnecessary killing of viable embryos. The same reason was also found in above mentioned US study. Regarding the use of PGD for choosing the gender of the baby, will change the original goal of PGD, which is having a healthier society into different personal goals. The fear of social pressure and self-blame, which could affect the initial intentions of the PGD candidates was expressed in a study conducted by Olesen et al. [18].

Limitations include being an online study the participants’ responses might not have been their own actual knowledge or perceptions and also it lacks the provision of enhancing the queries of the online questionnaire for some participants. Therefore, we recommend a face-to-face or telephone call interviews study to gather actual insight of people about this issue, which can help devising a strategy for pre-implantation genetic testing. Regarding the strength of this study, it is probably the first PGT study with an adequately large number of participants carried in Al Ahsa region of Eastern Province, KSA.

## Conclusions

The perception of Saudi citizens of Eastern Province toward PGT is assessed to be low. Increasing their perception toward such technology is needed as it is known that many chromosomal abnormalities are prevalent among this population, particularly sickle cell disease. The consistent finding of low perception but good acceptance of PGT in other aforementioned studies was found despite the availability of PGT in Saudi Arabia. Additionally, Saudis' opinions are almost influenced by cultural and religious points of view; care should also be given to this aspect. Achieving high awareness and positive perception towards PGT will eventually decrease the burden of many prevalent inherited diseases.

## Appendices

Section 1: personal information

This information is for research reasons only. They will not be used to identify you in any way.

1- What is your gender?

A. Male

B. Female

2- What is your age?

A. 18 - 20

B. 21 - 30

C. 31 - 40

D. 41 - 50

E. >50

3- What is your marital status?

A. Single

B. Married

C. Divorced

D. Widow

4- How many children do you have?

A. 0

B. 1

C. 2

D. 3

E. 4

F. More than 4

5- From which province of KSA are you?

A. Eastern province.

B. Western province.

C. Central province.

D. Northern province.

E. Southern province.

6- What is your approximate monthly income?

A. 5000 or less

B. 5,000 - 10,000

C. 10,000 - 15,000

D. 15,000 - 20,000

E. More than 20,000

7- Which of the following best describes your level of education?

A. Primary school degree

B. Middle school degree

C. Secondary school degree

D. Diploma degree

E. Bachelor’s degree

F. master

8- Are you a health care provider or a student in a medical field (medicine, nursing, pharmacy, applied medical sciences?

A. Yes. (If yes, please mention your specialty:…..)

B. No

9- Do you personally know anyone with a genetic or developmental disorder?

A. Yes

B. No

10- If yes, what is this genetic or developmental disorder?

A. Sickle cell anemia

B. G6PD

C. Down syndrome

D. No, I do not know anyone with a genetic condition or developmental disorder.

E. Other, please specify: ……

11- Do you personally require or know anyone who required the help of assisted reproductive technology to achieve a pregnancy?

A. Yes

B. No

Section 2: community awareness and acceptance

1- Prior to this study have you ever heard of preimplantation genetic diagnosis (PGD)?

A. Yes

B. No

To conduct the preimplantation genetic diagnosis "PGD", women should first undergo in vitro fertilization (IVF). IVF is where the woman's egg and the man's sperm are combined outside the human body in order to form a fertilized egg, which then grows into an embryo. In PGD, one or two cells from an embryo are removed and tested for various diseases. If a specific disease is identified, then the embryo is discarded. If there are no identified diseases, then the embryo is placed in the woman's uterus who will be pregnant with the ultimate goal of a healthy baby. PGD can detect diseases that are fatal in the first few years of life as well as diseases that can cause significant disabilities throughout a person's life.

In the following questions, please indicate the answer that best reflects your own personal opinion.

2- Doctors should be able to perform PGD to screen for diseases that are fatal in the first few years of life:

A. Agree

B. Neutral

C. Disagree

3- Doctors should be able to perform PGD to screen for diseases that cause lifelong disability such as mental retardation or deafness.

A. Agree

B. Neutral

C. Disagree

4- Doctors should be able to perform PGD to screen for diseases that may not occur until later in life, such as diseases that place an individual at a high risk of cancer during adulthood.

A. Agree

B. Neutral

C. Disagree

5- If you “agree” to questions 2, 3, or 4 that doctors should be able to perform PGD, which of the following statements best describes your reasons? (You can select more than one answer)

A. PGD improves the chances that a couple will have a healthy child

B. PGD will lower healthcare requirements and costs.

C. PGD can eliminate certain genetic diseases forever and may result in a better society.

D. It is acceptable according to the religious view.

E. Other, please specify: ……

6- If you “disagree” to questions 2, 3 or 4 that doctors should be able to perform PGD,

which of the following statements best describes your reasons? (Please select all that apply)

A. PGD leads to the unnecessary destruction of embryos after discovering having the genetic or

developmental disease.

B. PGD promotes discrimination against people with certain diseases

C. PGD interferes with nature and places doctors in the role of “playing God”

D. Widespread use of PGD may lead to unexpected consequences

E. It is unacceptable according to the religious view.

F. Other, please specify: ……

While PGD is a procedure that is most commonly used to identify diseases, in the future it potentially can be used to test for physical characteristics, personality traits, abilities, or sexual orientation.

Please indicate the answer that best reflects your own personal beliefs.

1- Doctors should be able to perform PGD for sex selection.

A. Agree

B. Neutral

C. Disagree

2- Doctors should be able to perform PGD to screen for physical characteristics such as height,

eye color.

A. Agree

B. Neutral

C. Disagree

3- Doctors should be able to perform PGD to screen for personality traits such as intelligence

or aggression.

A. Agree

B. Neutral

C. Disagree

4- If you “agree” to questions 1, 2, or 3 that doctors should be able to use PGD for the selection of ideal traits, which of the following statements best describes your reasons? (you can choose more than one answer)

A. Couples should be able to make their own decisions about their child

B. Selecting ideal traits will help a child lead a successful life

C. Selecting ideal traits will result in a better society

D. It is acceptable according to the religious view.

E. Other, please specify: ……

5- If you “disagree” to questions 1, 2, or 3 that doctors should be able to use PGD for the selection of ideal traits, which of the following statements best describes your reasons? (you can select more than one answer)

A. PGD leads to the unnecessary destruction of embryos.

B. PGD promotes discrimination against people with certain characteristics

C. PGD interferes with nature and places doctors in the role of “playing God”

D. Widespread use of PGD may lead to unexpected consequences

E. It is unacceptable according to the religious view.

F. Other, please specify: ………
